# Changes in Foliar Functional Traits of *S. pyrenaicus* subsp. *carpetanus* under the Ongoing Climate Change: A Retrospective Survey

**DOI:** 10.3390/plants9030395

**Published:** 2020-03-23

**Authors:** Rosina Magaña Ugarte, Adrián Escudero, Daniel Sánchez Mata, Rosario G. Gavilán

**Affiliations:** 1 Unidad de Botánica, Departamento de Farmacología, Farmacognosia y Botánica, Facultad de Farmacia, Universidad Complutense de Madrid, 28040 Madrid, Spain; dsmata@ucm.es (D.S.M.); rgavilan@ucm.es (R.G.G.); 2 Departamento de Biología y Geología, Física y Química Inorgánica, Universidad Rey Juan Carlos, 28933 Móstoles, Madrid, Spain; adrian.escudero@urjc.es

**Keywords:** stomatal traits, leaf morphology, high-mountains, herbarium collections, climate change

## Abstract

The sensitivity of stomatal behavior and patterning (i.e., distribution, density, size) to environmental stimuli, renders them crucial for defining the physiological performance of leaves. Thus, assessing long-term modifications in stomatal traits in conserved specimens arises as a valuable eco-physiological approach to predict how the rising trend of warmer, drier summers could affect plant fitness; particularly in mountain areas already experiencing climate aggravation and lacking the related monitoring schemes like Mediterranean high-mountains. Variations in foliar and stomatal traits were studied in conserved specimens of *Senecio pyrenaicus* subsp. *carpetanus* from Sierra de Guadarrama over the past 71 years. Our findings revealed decreasing trends in leaf width, stomatal size, and increasing tendency in stomatal density, all correlated with the recent 30-year climate exacerbation in these mountains. This evidenced a positive selection favoring traits that allow safeguarding plant performance under drier, hotter weather conditions. The significant relation between stomatal traits and climatic variables upholds the role of stomatal patterning in sensing environmental cues in this species, feasibly optimizing physiological responses involved in the growth–water loss trade-off. The transition to smaller, densely packed stomata observed in recent decades could indicate local-adaptive plasticity in this species, enhancing stomatal response, as coarser environmental conditions take place in Sierra de Guadarrama.

## 1. Introduction

In terrestrial ecosystems, plants provide an array of services that constitute the basis for the sustainability and long-term dynamics of ecosystems; besides driving their ability to respond to disturbances [1]. Therefore, the assessment of universally appropriate predictors of ecosystem function and responsiveness to change is critical, especially in view of the ongoing biodiversity and climate crises imperiling our ecosystems [2,3]. Knowledge and identification of these plant functional attributes, associated with primary strategies (i.e., growth, resource gaining, reproduction), could grant improved predictions of the responses of plant species, and in turn that of plant communities, to impending disturbances in their environments [4,5]. For instance, appraisal of functional leaf traits could help identify adaptive features on leaf architecture linked to carbon gain, nutrient loss, and water usage in plants. This will enable evaluating changes in plant performance over time, inferring population dynamics and, by extension, community assembly in the face of changing environmental conditions [5]. Particularly, if properly selected, these plant functional attributes and their time-related changes could inform on the functional plasticity of plant populations responding to environmental fluctuations. 

Experiments under controlled environments, though important for studying plant responses to changing levels of environmental factors, are unable to reproduce the effects of these changes through prolonged periods or involving numerous generations [6,7]. Thus, the study of longstanding plant responses to environmental wavering requires for observational and long-term monitoring schemes, which are infrequently feasible approaches given the scarcity of established surveying programs within the past century, especially in hardly accessible areas such as high-mountains. A rarely explored alternative is the indirect approach of studying the long-term variations and trends of plant traits via the use of historic leaf herbarium specimens [6,8,9]. Provided the studied traits are adequately preserved in the conserved specimens, assessment of herbarium collections offers a valuable tool not only to study long-term distributional, morphological, and phenological variations of species across spatial scales; but also, to reproduce long term phenotypic variations along shifting climates when monitoring programs are inexistent [7].

Stomata play a key role in determining the trade-off between growth and water conservation in plants [10]; having the rate at which a gas diffuses through the stomatal aperture (stomatal conductance, *g_s_*) ultimately limited by the number and size of stomata on the leaf epidermis [11]. Consequently, stomatal distribution, density, and regulation govern the CO_2_ and water exchange rates. Since these have a direct effect on the photosynthetic carbon assimilation to water-loss ratio or evaporative cooling, they become key attributes in the definition of the physiological potential and photosynthetic performance of leaves [6,10,12,13]. Stomatal behavior and patterning (i.e., distribution, density, size) respond to a series of environmental factors and plant signals. Similarly, changes in leaf morphology can be induced by climate gradients, such as those prevailing in high-mountain areas; especially in broad-leaved species given their higher liability to climatic variations [14,15]. Seeing as species-specific adjustments in stomatal density (number of stomata per mm^2^, SD) and anatomy (i.e., stomatal size, SS) are unequivocally linked with ecological heterogeneity, studying their variation alongside other leaf morphological traits could expound acclimation responses of species [12,16,17]. Furthermore, long-term anatomical adjustments in SD and SS ultimately target an improved stomatal sensitivity and faster adjustment of aperture in response to changing environments, thus having an effect on the cost-effectiveness of photosynthesis [18]. In turn, assessing modifications in stomatal patterning and structures in leaves from conserved specimens (a morphological trait well conserved in ex situ collections for prolonged periods) provides insight on the conditions and plant responses at the time of collection [19,20]. Additionally, given the strong link between stomata and water loss in plants, evaluating stomatal patterning modifications through time in collected individuals could also enable unveiling the response to impending warmer and drier growth seasons, particularly in water-limited environments such ass Mediterranean high-mountains [12,16,21,22]. Currently available studies on stomatal morphology, its long-term variation, and ecophysiological connotations are limited to lowland or woody species and rarely involve herbaria specimens; while their study in high-mountain herbaceous vegetation remains somewhat scant and focused on plants from mountains with temperate climates [12,17,23,24]. To our knowledge, the information accumulated in herbaria through years of systematic collection of populations has been scarcely regarded and considered for describing variations along gradients [25] or in response to shifting climate [9,23,26]. 

In the high-mountains of Mediterranean-type climate, such as the Sierra de Guadarrama in Central Spain, the greatest constraint for plant growth is summer drought, co-occurring with high temperatures and irradiance during the short active growing season of vegetation. Climate records have advocated for a general annual shift towards drier conditions since the 1990s, coupled to an increase in summer warming overlapping with the brief active-growing season [27,28]. In turn, physiological and morphological adaptations are impending in plants inhabiting these areas to counteract the constraining environmental conditions befalling the brief window for plant activity. *Senecio pyrenaicus* subsp. *carpetanus* (hereafter ‘*S. carpetanus’*) is a frequent species of the pioneer plant community thriving in stony, siliceous, mobile screes in areas above the tree line in Sierra de Guadarrama. Its coverage surges alongside increased soil consolidation and integrity in any stony soil, fostering the gradual establishment of species proper of these psicroxerophitic grasslands [29]. To date, studies on long-term changes in leaf anatomical traits in Mediterranean orophytes through assessment of herbarium specimens are inexistent, similar to the lack of studies relating longstanding modifications in stomatal and foliar traits with climatic factors, despite the critical demand existing to unveil the response of these species to the ongoing climate warming. Albeit the absence of programs monitoring the long-term variations in the aforementioned traits in Mediterranean high-mountain orophytes, their study is feasible on account of their existing herbarium collections. The assessment of these herbarium collections offers a valuable tool to reproduce the adjustments in these orophytes along wavering climates and, thus, enable reconstructing their plastic responses in stomatal and foliar traits to shifting conditions, within the context of climate change. 

Assessing the real effect of climate aggravation on the species conforming Mediterranean high-mountain communities is critical for their conservation, since they shelter an important number of endemics and high-mountain specialists reaching their southernmost limit in these mountains [30]. The latter is further exacerbated given the restricted possibility of these orophytes to migrate to avoid the constraints of climate change as a result of the relatively small altitudinal range of these mountains (e.g., warming, increased species competition with lower-altitude and upcoming species); and further precluded by the E–W orientation of Sierra de Guadarrama, impeding extending their ranges to higher latitudes [30,31,32]. In order, the persistence of these orophytes in these mountains ought to be driven by their ability to overcome the effects of climate exacerbation, via either local-adaptations or phenotypic plasticity in traits related to primary strategies. Consequently, and given the nonexistence of related monitoring programs, we evaluated the potential variations in morpho- and micro-morphological leaf traits in herbarium specimens of *S. carpetanus* inhabiting the highest summits of Sierra de Guadarrama through the last 72 years, expecting to reveal possible adjustments in leaf morphology, SD and SS. In accordance, we hypothesize that (1) these potential adjustments in leaf macro and micro-morphological traits occurred in function of the reported aggravation of climatic conditions during the brief growing season (summer period) in Sierra de Guadarrama, as an outcome of climate change [28]. Or (2) advocate the observed changes should be regarded as monotonic, meaning they only vary as a response to the environmental gradients characteristic of high-mountains.

## 2. Results

**Figure 1:**
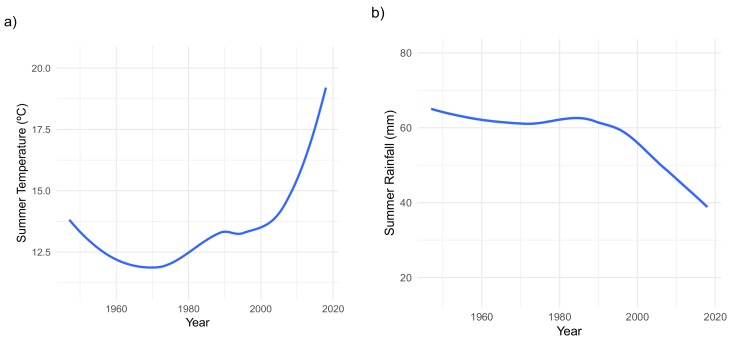
(**a**) Modeled data for temperature variations during the summer season in Mediterranean high-mountains of Sierra de Guadarrama (i.e., active growing season of *S. carpetanus*) between 1946 and 2018; (**b**) modeled trend of the rainfall patterns befalling the summer season during the last 72 years in Sierra de Guadarrama high-mountain areas. In both graphics, the blue line corresponds to the modeled data; the gray area represents the “smoothed” parameter estimation.

Analysis of climatic records for the period 1946–2018, indicate an increasing tendency towards warmer summers and winter seasons in these mountains in the last 10 years (2009–2018; Figure 1a,b). In addition, we detected a decreasing trend in annual rainfall in the last decade. These data records also reflect an abrupt shift towards drier conditions in Sierra de Guadarrama mountains as from the 1980s.

### Morphological and Micromorphological Parameters

**Figure 2:**
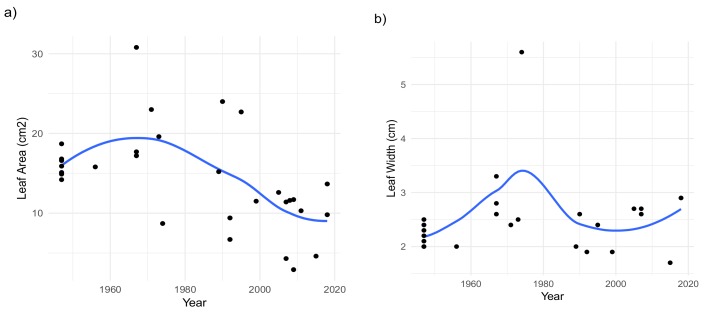
Generalized additive models for (**a**) changes in leaf area in *S. carpetanus* from the high-mountain habitats of Sierra de Guadarrama in the last 72 years; (**b**) leaf width variations in conserved specimens of *S. carpetanus* over the last 72 years. The blue lines correspond to the modeled data; the gray area represents the “smoothed” parameter estimation, in accordance to the generalized additive models (GAMs).

Our analyses revealed a significant reduction in leaf width under years with lower rainfall during the growing season (*p* value= 0.02), whilst denoting higher values in samples from specimens growing under wetter conditions. The latter refers to samples from the period 1956–1973, where the sourced specimens possessed the highest values for leaf width, associated with the wetter and cooler conditions registered (Figure 2b). Moreover, the tendency of rising temperatures recorded from the 90s and onwards, had a significantly negative effect (*p* value = 0.03).

A significant reduction in leaf area (LA) was observed in specimens from the last 18 years, subject to the significantly higher temperature and low rainfall conditions (*p* value < 0.001, for both parameters) befalling the active growing season (Figure 2a, Figures S2 and S3). In addition, the negative effect of reduced precipitation during the growing season was more significant in samples from the last three decades, compared to those from earlier dates (*p* value < 2 × 10^−16^ and =1.29 × 10^−6^, respectively).

**Figure 3:**
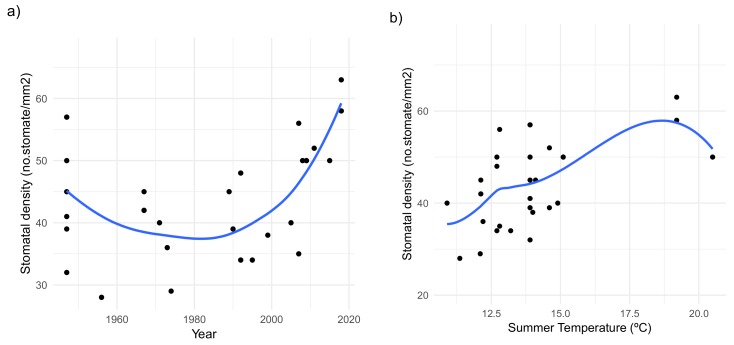
Generalized additive models for (**a**) changes in stomatal density over the last 72 years in *S. carpetanus* conserved specimens from Sierra de Guadarrama mountains; (**b**) stomatal density fluctuations in response to mean daily temperatures during the active growing season (summertime) on *S. carpetanus* conserved specimens. Blue lines fit the modeled data, while the immediate gray area denotes the “smoothed” parameter estimation compliant with the GAMs.

The significant effect of higher temperatures befalling the growing season in recent years over SD was reflected in the increasing trend of this trait in samples from 1990 to 2018 (Figure 3a,b); whilst denoting a significant reduction when subject to a colder, wetter growing season (1956–1973). Seasonal rainfall patterns, either during the wet or dry season, showed to have no overall significant effect on SD. A significant increase in SD was observed in samples before the 90s, while no significant differences were observed among those from the last 28 years. 

**Figure 4:**
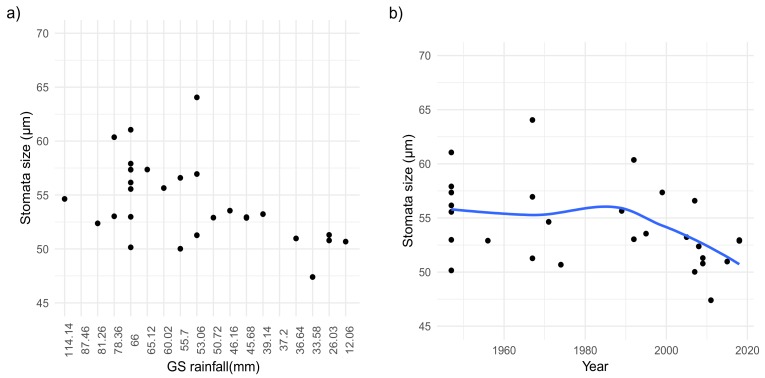
(**a**) Variations in stomatal size in response to rainfall regimes during the growing seasons throughout 72 years in *S. carpetanus* from Sierra de Guadarrama mountains; (**b**) overall variation in stomatal size in conserved specimens of *S. carpetanus* from Sierra de Guadarrama between 1946 and 2018. Blue lines represent the modeled data and the surrounding gray area indicates the estimated variation of the parameter, according to the GAMs.

Additionally, our results suggest the plausible association between the observed shrinkage trend of SS and the registered decreasing rainfall patterns during the growing season over the last 20 years (Figure 4a,b). The latter given the reduced SS found in individuals collected under growing seasons with rainfall below normal values, expressly in 2009 and 2011 registered as two of the driest growing seasons in these mountains within the time of our study. 

The smaller SS was particularly notable for individuals from the 90s and onwards which displayed a significant reduction in SS in response to the overall decline in annual precipitation regimes observed in Sierra de Guadarrama [27]. Moreover, the higher temperatures befalling the active vegetation period in recent years also had a significant effect over the variation in SS (*p* value = 0.02).

## 3. Discussion

In plants, growth strategies are commonly related with specific habitat adaptations, primarily in herbaceous species with shorter life spans [33]. Variations in SD and SS can be the result of the effect of genetic and environmental factors to which the plant is subject during its growing season [18]. However, the rapid rate of the modern climate change could be a greater crucial driver of the ecological response of plants, compelling them to display local plant trait variations in order to buffer the effects of such change and persist in their habitats [34]. The latter is particularly plausible in areas enclosing steep environmental gradients within short distances, such as high-mountains, where the expression of a single phenotype by a species will unlikely grant proper fitness under the diversity of environmental settings varying at short scales in these habitats [15,34,35]. In turn, life history results in a vital factor shaping trait differentiation and functional ecology of “subordinate” species as *S. carpetanus*, able to thrive in similar niches through a variety of habitats granting them a high degree of homeostasis when exposed to fluctuating or constraining settings [4,33]. 

Under cooler conditions, plants can foster the development of bigger leaves to heighten their boundary layer thickness, allowing a faster attainment of the propitious temperatures for photosynthesis [36]. The greater LA observed in conserved specimens of *S. carpetanus* that experienced continual cold, wet growing seasons between 1956 and 1973 (Figure 1b and Figure 2a; [27]) contrasts the response of those specimens collected in years with drier, hotter growing seasons. This suggests the ability of *S. carpetanus* to adjust its leaf macro-morphology to potentially maximize photosynthetic returns in temperate growing seasons through improved heat exchange capacity as an outcome of the greater boundary layer thickness in bigger leaves [36]. 

In context of the ongoing rising of global temperatures, plants not only need to reduce water loss but also tackle leaf overheating to prevent potential photoinhibition, leaf damage, and yield reductions. In turn, this could lead to higher SD and possibly increased *g_s_*, enabling overcoming heat stress through increased transpiration-mediated cooling [18]. The significant effect of higher seasonal temperatures on LA could justify the decreasing pattern observed in this trait within the past three decades in *S. carpetanus* conserved specimens (Figure 2a and Figure S2a). These findings allow suggesting the observed shift towards smaller leaves as a response in this species to avoid photosynthetic impairment caused by the latent inefficient convective heat-transfer in bigger leaves under drier, hotter climates [37]. Additionally, the significant decline in SD observed in conserved specimens from years with milder growing seasons contrasts the densely-packed, smaller stomata in *S. carpetanus* individuals from recent decades growing under drier, hotter conditions (Figure 3a,b and Figure S1). The latter indicates the proposed morphology–fitness relationships being context–driven, i.e., responding to environmental conditions present in each growing season. 

The concurrent decrease in LA and SS, coupled to higher SD in *S. carpetanus* individuals in response to the latest 30-year climate exacerbation in the Sierra de Guadarrama summits, provides evidence of positive selection favoring traits that allow safeguarding plant performance under drier, hotter weather in this species [38]. Similar findings in different species experiencing concomitant warming and drought, support the proposed existence of a feasible uniform trait response across phylogenetic scales in plants habituated to recurrent dry growing seasons [38,39]. Correspondingly, our findings further contribute to underlining the importance of examining local plant trait variations for indicating the ability of plants to adapt to wavering environmental factors, aiding to outline their vulnerability to the foreseen climate exacerbation [34,40]. 

The strong and significant relations found between stomatal traits and climatic variables befalling the growing season (i.e., temperature, rainfall) put forward the implication of stomatal patterning in the local acclimatization of *S. carpetanus*, feasibly optimizing the physiological responses involved in the growth–water loss trade–off of this species. Similar to that observed in alpine herbs [23], our study revealed the significant effect of warmer growing seasons on SD accompanied by the development of smaller stomata in *S. carpetanus*, particularly in recent years. Concurring with recent studies in this species [41], the present findings advocate for an enhanced ecological tolerance in *S. carpetanus* to the governing stress factors during the growing season in Sierra de Guadarrama. Moreover, our results also resemble those of Carlson et al. [38], Xu and Zhou [22] indicating an increased SD and reduced SS as an enhancement of drought tolerance in plants experiencing dry growing seasons in their natural habitats. The transition to smaller, densely–packed stomata in *S. carpetanus* could indicate a local–adjustment strategy in this species to optimize stomatal response, coupled to a potential improvement of water–use–efficiency (WUE), as coarser environmental conditions take place. Nevertheless, it is worth noting that the implemented approach of assessing general trends in these plant and stomatal traits limits our power for pinpointing whether these reflect adaptive processes (i.e., evolutive) or merely phenotypic plasticity. 

Inferring ecological trends as the stronger driver for regulating behavior of stomata [17,42], the observed effects of the long-term exposure of *S. carpetanus* to gradual increases in water deficit during the growing season denotes an apparent higher resilience capacity to the forthcoming climate aggravation in Mediterranean high-mountains. Moreover, the association among reduced SS and, consequently, higher δ ^13^C with historic drought regimens in Spanish accessions of *Arabidopsis thaliana* [43], provides additional evidence inferring the observed adjustments in stomatal morphology in *S. carpetanus* as the outcome of the ecological trends recorded in the last three decades, i.e., the aggravation of climate in the growing season. However, further studies incorporating photosynthetic and stomatal regulation parameters, added to nutrient status, are encouraged to determine whether these adjustments are sufficient to sustain efficient photosynthetic activity. Incorporation of these analyses will provide a holistic understanding of the ecophysiology in *S. carpetanus*, coupled to accurately assessing the vulnerability of the structure and dynamics of these high-mountain communities, in the context of climate change. 

As postulated, the use of specimens preserved in historical collections may contribute to evaluating the long-term responses of key leaf traits linked to the adjustment capacity of high-mountain plants to shifting climatic conditions, the latter considering that alterations in the frequency of certain plant traits are anticipated as consequence of wavering climate [44]. Consequently, these findings support them as practical tools to monitor wild populations of plant species and assess the impact of future climate change upon biodiversity in habitats highly vulnerable to the effects of climate change, such as Mediterranean high-mountains. More to the point, the use of conserved specimens could translate into an upgraded ability for reconstructing past plant behavior in these habitats with actual evidence of the latter and where monitoring schemes are unavailable. What is more, the outcomes of such studies could be indicative of the sensitivity of these orophytes to the expected coarser conditions in Mediterranean high-mountains, as consequence of the ongoing climate crisis. 

## 4. Materials and Methods 

### 4.1. Study Area

**Figure 5:**
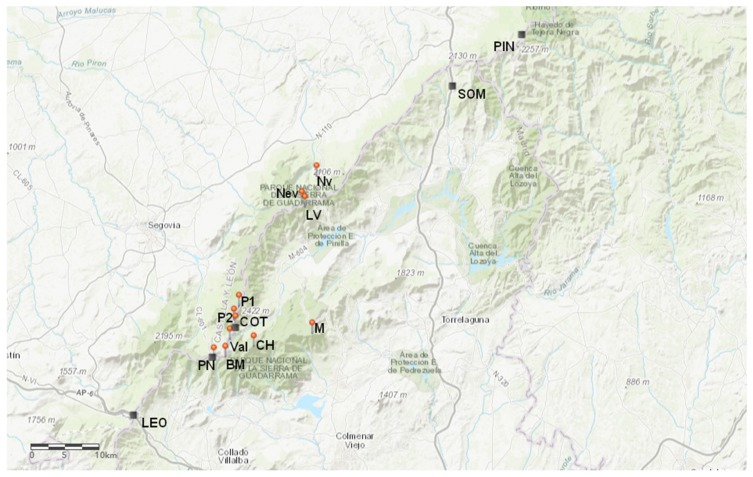
Location of sampling points (red points) and meteorological stations (black squares) along the Sierra de Guadarrama mountain range in Central Spain. BM, Bola del Mundo; CH, Cabeza de Hierro; COT, Puerto de Cotos; LV, Lozoya del Valle; LEO, Alto del León; M, Puerto de la Morcuera; PN, Puerto de Navacerrada; Nv, Navafría; Nev, El Nevero; P1, Peñalara 1; P2, Peñalara 2; PIN, La Pinilla; SOM, Somosierra; Val, Valdesquí.

We selected plant material from populations from the Sierra de Guadarrama range, a segment of the Sistema Central mountain range in central Spain. The chosen area corresponds to the optimal altitude distribution range of *S. carpetanus* in the region (Figure 5; [29]). The Sierra de Guadarrama range experiences a Mediterranean-type climate: cold temperatures during winter, and elevated temperatures added to limited rainfall during summer. Mean annual rainfall is ≈1350 mm, with a summer-drought period going from May to October that accounts for <10% total annual rainfall. The topsoil water content (10 cm) measured in these summits as part of the GLORIA project reads of <5% in August and <10% in September, translating into reduced water availability during the active growing season of high-mountain flora [45].

### 4.2. Plant Material

*Senecio pyrenaicus* is a highly variable perennial herb, with several variants among the Spanish mountains. The *S. pyrenaicus* subsp. *carpetanus* (Willk.) Rivas-Mart. (from now ‘*S. carpetanus’*) growing in Sierra de Guadarrama is a perennial herb regarded as the dominant species on stony, siliceous mobile screes [29]. It finds its altitude optimum above 1800 m; with its emergence strongly related to snow melting time. *S. carpetanus* reaches heights between 20 and 45 cm, with erect, densely leafed stems that only branch in inflorescence [46].

Herbarium collections of this subspecies were sourced for locations of the highest summits in Sierra de Guadarrama. Specimens studied (at least one per year assessed) comprised those herbarium specimens collected between the period 1947–2018 and within 1773–2428 m.a.s.l. (Figure 5). The *S. carpetanus* specimens were kindly provided by MAF Herbarium (UCM, Madrid, Spain), SALA Herbarium (University of Salamanca, Salamanca, Spain), the Herbarium of the Universitat de Valencia (University of Valencia, Valencia, Spain), the RJB Herbarium (Real Jardín Botánico, Madrid, Spain), and CIEMAT (Centro de Investigaciones Energéticas, Medioambientales y Tecnológicas, Madrid, Spain). In addition, we collected new, fully developed individuals in recent years during the growing season in these mountains (summertime) at 2244 m, consisting of fully developed individuals. These were pressed and handled as herbarium sheets in order to have all assessed samples under similar conditions. A list of all specimens and their respective location is provided in Table S1.

### 4.3. Stomatal and Other Leaf Traits

Assessment of stomatal features was performed on fully developed leaves of conserved specimens. Leaf imprints were made following the silicone rubber technique described by Weyers and Johansen [47], using impression material (A-Silicone Putty Fast Set. VANINI Dental Industry, Grassina, FI, Italy), and image analysis was carried out as described by Fanourakis et al. [48]. Leaf rubber impressions were taken from the mid–portion of the abaxial surface of the leaves, avoiding the main vein. A total of 20 stomata per leaf surface were randomly chosen to determine stomatal length and width. SD and SS were determined in five non–overlapping fields of view per imprint using a Nikon (ECLIPSE 80i) digital microscope attached to a camera control unit (DS Camera Control Unit DS-L2, Nikon Metrology Inc.; Nikon Metrology GmbH Alzenau, Germany).

General leaf morphological traits were also measured on all conserved specimens according to the protocols established by Cornelissen et al. [49]. The leaf length and width, plant height, and leaf area were measured in all samples, on dry fully developed leaves. LA was calculated using Image J free image analysis software (ImageJ, U. S. National Institutes of Health, Bethesda, MD, USA), from scanned dry and fresh leaves. For specimens collected in 2018, plant height measurements were taken in situ on fully developed individuals. Fully developed fresh leaves were collected for LA measurements, following their pressing and drying to obtain the corresponding dry LA.

### 4.4. Climate Data and Statistical Analysis

Data of 28 climatic descriptors for the Sierra de Guadarrama summits was obtained from the meteorological station Puerto de Navacerrada, Madrid (ran by the Spanish Institute of Meteorology), which were used to characterize each year. In order to select the most relevant climatic descriptors, we performed PCA analysis using the packages “stats”, “Factoextra”, and “FactoMineR” [50,51,52]. In this paper, the “dry season” refers to the summer stress interval (May–September), befalling the active growing season in Mediterranean high-mountains; and the “wet season” denotes the period with higher rainfall and snowfall in the area of study (October–April).

In order to determine the effect of these climatic descriptors on changes in LA, leaf width, SD and SS, we performed generalized additive models (GAMs) using the data collected from the conserved samples (1947–2018) using the “mgcv” package [53]. The initial models for all traits included the additive effect of the climatic variables determined as relevant in the PCA (i.e., mean annual temperature, mean annual rainfall, dry season average temperature, dry season average rainfall), and the additive effect of altitude as predictors. Moreover, based on studies indicating an increasing tendency in drier, hotter conditions in these mountains as from the 90s [27,28], the data was further subdivided into two groups (1) samples before 1990; and (2) samples from 1990 and onwards. These subsets were also analyzed with GAMs, maintaining the same factors as in the overall first analyses. 

Prior to the GAM analyses, we tested for multicollinearity among predictors assessing the variance inflation factor (VIF) with the “vif” function of the “car” package [54]. Given maximum VIF values were lower than three, all variables were included in the initial models. 

All statistical analyses were performed with the R statistical software [51].

## Supplementary Materials

The following are available online at https://www.mdpi.com/2223-7747/9/3/395/s1. Figure S1: Mean SD variation in *S. carpetanus* samples from the summits of Sierra de Guadarrama in response to the increasing annual temperatures over the last 72 years (1946–2018). The blue line represents the data and the surrounding gray area indicates the estimated variation in the response in compliance with the GAMs; Figure S2: Generalized additive models for (a) changes in leaf area in *S. carpetanus* from the high-mountain habitats of Sierra de Guadarrama in response to the increasing temperatures during the growing season over the last 72 years; (b) changes in the leaf area on conserved specimens of *S. carpetanus* over the last 72 years as a function of variations in the rainfall regimes incident during its growing season (summertime). The blue lines correspond to the modeled data; the gray area represents the “smoothed” parameter estimation, in accordance to the GAMs; Figure S3: Generalized additive models for the variations in leaf width of *S. carpetanus* leaves collected through 1946–2018 in response to the decreasing rainfall patterns during its growing season in Sierra de Guadarrama; Table S1: Herbaria data of the studied *S. carpetanus* specimens selected for the present study, coupled with the mean morphological parameters measured per herbarium record. Herbaria data includes the respective location and information of each consulted sheet. Significant effects of summer temperature and rainfall are represented by asterisks: ***, *p* < 0.001; **, *p* < 0.01; *, *p* < 0.05.
