# Supplementary material for: Changes in Foliar Functional Traits of S. pyrenaicus subsp. carpetanus under the Ongoing Climate Change: A Retrospective Survey
Source: Plants (Basel). 2020 Mar 23;9(3):395. doi: 10.3390/plants9030395 (PMC7154879; doi:10.3390/plants9030395)
Supplement: Supplementary file 1 [file plants-09-00395-s001.zip › Supplementary files/Supplementary Material, foliar morphology S.carpetanus.docx]

**Table S1.** Herbaria data of the studied *S. carpetanus* specimens selected for the present study, coupled with the mean morphological parameters measured per herbarium record. Herbaria data includes the respective location and information of each consulted sheet. Data is presented in mean values per individual assessed. Significant effects of summer temperature and rainfall are represented by asterisks: ***, *P <* 0.001; **, *P < 0.01*; *, *P < 0.05*.

| Herbarium/ Institution | Herbarium ID | Year | Sampling point | No. stomate | Stomate length  (µm) | Stomate width (µm) | Stomatal density (no.stomate/mm^2^) | Std dev. | Leaf Area (cm^2^) | Plant height (cm) | Leaf width (cm) | Leaf length (cm) |
| --- | --- | --- | --- | --- | --- | --- | --- | --- | --- | --- | --- | --- |
| MAF/ Universidad Complutense de Madrid | MAF 79092 | 1947 | Peñalara | 61 | 50.2 | 40.6 | 57 | 6.3 | 15.9 | 34.1 | 2.3^*^ | 6.7 |
| SALA/ Universidad de Salamanca | SALAF 3139 | 1947 | Peñalara | 54 | 53.0 | 41.8 | 50 | 1.3 | 18.7 | 40.1 | 2.1^*^ | 6.9 |
|  | SALAF 3144 | 1947 | Peñalara | 62 | 56.2 | 40.4 | 57 | 2.1 | 14.9 | 32.1 | 2.0^*^ | 5.9 |
|  | SALAF 3149 | 1947 | Peñalara | 35 | 61.0 | 40.5 | 32 | 2.1 | 16.6 | 30.1 | 2.0^*^ | 6.2 |
|  | SALAF 3154 | 1947 | Peñalara | 49 | 57.9 | 39.5 | 45 | 6.5 | 14.2 | 20.1 | 2.4^*^ | 5.3 |
| Herbarium of the Universitat de Valencia | VAL 130497 (= VALF 2473) | 1947 | Peñalara | 44 | 55.6 | 37.9 | 41 | 6.0 | 16.8 | 30.0 | 2.2^*^ | 6.7 |
|  | VAL 130497 (= VALF 2473) | 1947 | Peñalara | 43 | 57.4 | 41.9 | 39 | 4.0 | 15.1 | 20.1 | 2.5^*^ | 5.2 |
| MAF/ Universidad Complutense de Madrid | MAF 120334 | 1956 | Bola del mundo | 30 | 52.9 | 37.0 | 28^*^ | 4.9 | 15.8 | 24.6 | 2.0 | 7.1 |
|  | MAF 69199 | 1967 | Navafria | 46 | 47.0 | 34.6 | 42^*^ | 4.1 | 17.7 | 33.8 | 2.8 | 5.7 |
|  | MAF 102378 | 1967 | Navafria | 49 | 64.0 | 53.6 | 45^*^ | 2.9 | 17.2 | 36.7 | 2.6 | 6.2 |
|  | MAF 152975 | 1971 | Cabeza de hierro | 43 | 54.6 | 42.6 | 40^*^ | 2.1 | 23.0 | 59.1 | 2.4 | 7.5 |
|  | MAF 115664 | 1973 | Peñalara | 39 | 39.8 | 32.3 | 36^*^ | 1.8 | 19.6 | 36.8 | 2.5 | 6.0 |
|  | MAF 119859 | 1974 | Navacerrada | 32 | 50.7 | 37.7 | 29 | 1.2 | 8.7 | 31.4 | 5.6^*^ | 1.9 |
|  | MAF 134292 | 1989 | Peñalara | 49 | 55.6 | 38.6 | 45 | 1.7 | 15.2 | 37.2 | 2.0^*^ | 5.7 |
|  | MAF 146691 | 1990 | Peñalara | 42 | 64.9^*^ | 57.6^*^ | 39^*^ | 1.2 | 24.0 | 34.1 | 2.6^*^ | 7.4 |
|  | MAF 144434 | 1992 | Valdesqui | 36 | 53.0^*^ | 43.3^*^ | 34^*^ | 1.4 | 9.4 | 49.0 | 1.9^*^ | 4.4 |
| CSIC-Real Jardín Botánico. Colection of Vascular Plants (MA) | MA 515384 | 1992 | Peñalara | 52 | 60.6^*^ | 36.7^*^ | 48^*^ | 1.6 | 6.7 | 52.0 | 1.2^*^ | 6.5 |
| Herbarium of the Universitat de Valencia | - | 1995 | Pto. Cotos | 36 | 53.5^*^ | 40.3^*^ | 34^*^ | 5.5 | 22.7 | 82.6 | 2.4^*^ | 6.6 |
| MAF/ Universidad Complutense de Madrid | MAF 157077 | 1999 | Peñalara | 41 | 57.4^*^ | 44.2^*^ | 38^*^ | 2.3 | 11.5 | 30.9 | 1.9^*^ | 5.1 |
| CSIC-Real Jardín Botánico. Colection of Vascular Plants (MA) | MA 750233 | 2005 | Puerto de la Morcuera | 44 | 53.7^*^ | 42.3^*^ | 40^**^ | 2.6 | 12.6^***^ | 25.1 | 2.7^*^ | 6.6 |
|  | MA 773457 | 2007 | Cumbre el Nevero | 38 | 56.1^*^ | 39.3^*^ | 35^**^ | 3.9 | 11.4^***^ | 31.9 | 2.7^*^ | 7.8 |
| CIEMAT | - | 2007 | Bola del mundo | 60 | 50.0^*^ | 37.7^*^ | 56^**^ | 1.9 | 4.3^***^ | 33.9 | 2.6^*^ | 9.4 |
|  | - | 2008 | Bola del mundo | 54 | 52.4^*^ | 40.2^*^ | 50^**^ | 1.1 | 11.6^***^ | 35.1 | ND | ND |
|  | - | 2008 | Bola del mundo | 56 | 47.0^*^ | 39.7^**^ | 52^**^ | 3.9 | 11.6^***^ | 30.2 | ND | ND |
|  | - | 2009 | Bola del mundo | 54 | 51.3^**^ | 40.^**^ | 50^**^ | 4.4 | 11.7 ^***^ | 33.2 | ND | ND |
|  | - | 2009 | Bola del mundo | 62 | 52.6^**^ | 40.0^**^ | 57^**^ | 2.0 | 2.9 ^***^ | 30.6 | ND | ND |
|  | - | 2011 | Bola del mundo | 60 | 51.0^**^ | 37.9^**^ | 56^**^ | 4.0 | 10.3^***^ | 31.4 | ND | ND |
| CSIC-Real Jardín Botánico. Colection of Vascular Plants (MA) | MA 906955 | 2015 | Lozoya del Valle | 76 | 57.9^*^ | 38.2^*^ | 70^**^ | 4.0 | 4.6^***^ | 26.7 | 1.7 ^*^ | 5.2 |
| MAF/ Universidad Complutense de Madrid | MAF 178101 | 2018 | Bola del mundo | 47 | 57.7^*^ | 40.3^*^ | 43^**^ | 2.4 | 9.8 ^***^ | 58.5 | 2.9^*^ | 8.0 |
|  | MAF 178101 | 2018 | Bola del mundo | 50 | 60.7^*^ | 41.4^*^ | 47^**^ | 2.5 | 13.7 ^***^ | 49.8 | 2.9 | 8.1 |
