# Supplementary figures and images for: Changes in Foliar Functional Traits of S. pyrenaicus subsp. carpetanus under the Ongoing Climate Change: A Retrospective Survey
Source: Plants (Basel). 2020 Mar 23;9(3):395. doi: 10.3390/plants9030395 (PMC7154879; doi:10.3390/plants9030395)

b)

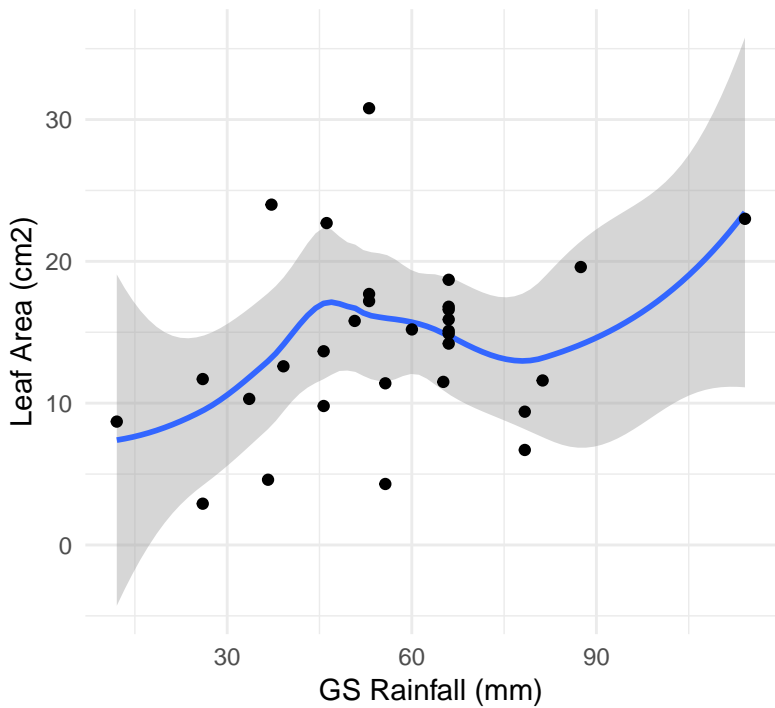

Supplement: Supplementary file 1 [file plants-09-00395-s001.zip › Supplementary files/Supplementary Figure 2b.pdf]

a)

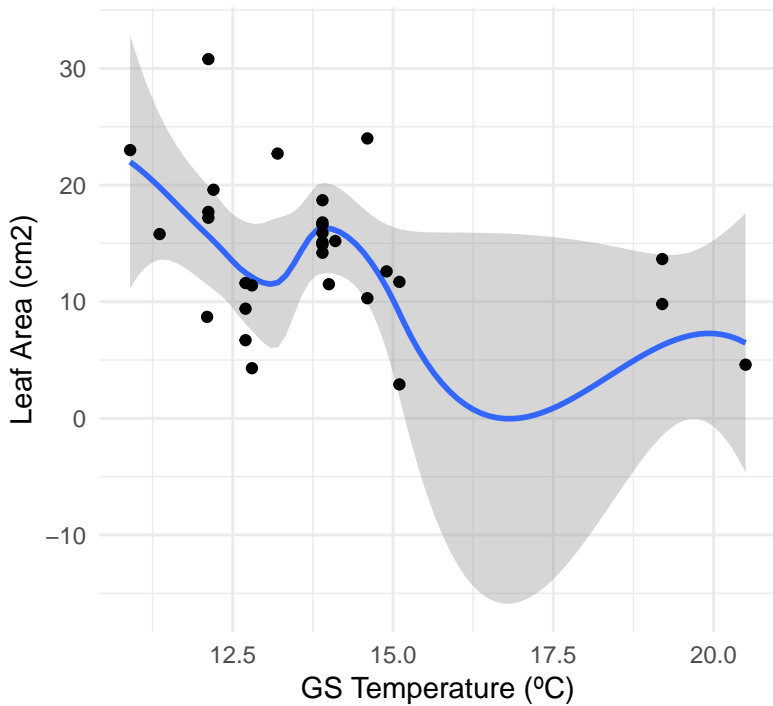

Supplement: Supplementary file 1 [file plants-09-00395-s001.zip › Supplementary files/Supplementary Figure 2a.pdf]

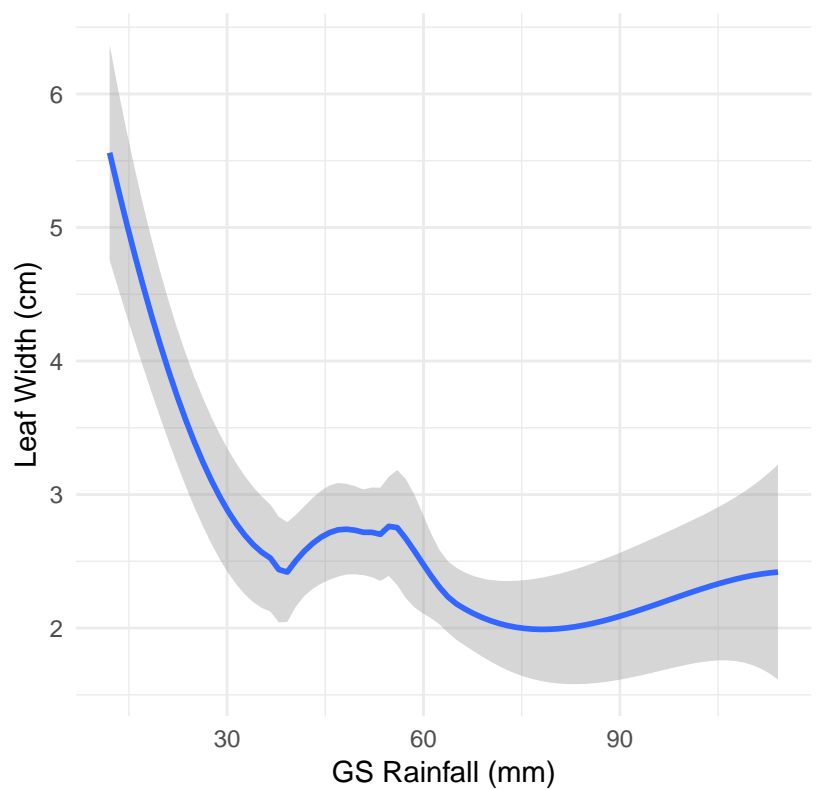

Supplement: Supplementary file 1 [file plants-09-00395-s001.zip › Supplementary files/Supplementary Figure 3.pdf]

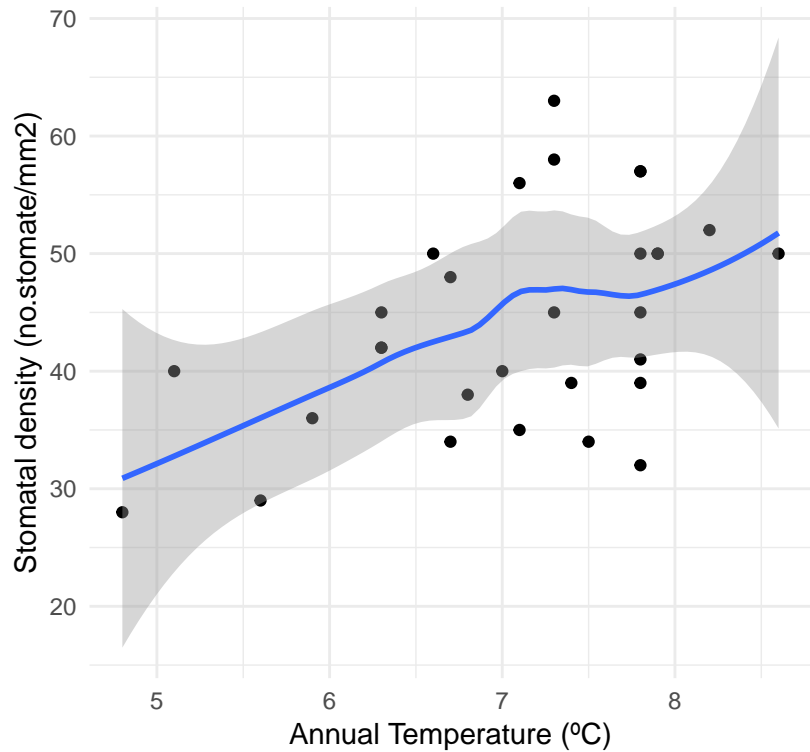

Supplement: Supplementary file 1 [file plants-09-00395-s001.zip › Supplementary files/Supplementary Figure 1.pdf]
